# Oehlers′ Type II Dens Invaginatus Case Report: Outcome of Sequential Management With Primary Targeted Invagination Treatment

**DOI:** 10.1155/crid/5528956

**Published:** 2026-07-17

**Authors:** Igor Noenko, Myroslav Goncharuk-Khomyn, Mariya Kubatska

**Affiliations:** ^1^ Private Practice, Kyiv, Ukraine; ^2^ Associate Professor of Restorative Dentistry, Faculty of Dentistry, Uzhhorod National University, Uzhhorod, Ukraine; ^3^ Private Practice, Gdansk Medical University, Gdansk, Poland, mug.edu.pl

**Keywords:** case report, dens-in-dente, endodontics, root canal preparation, success

## Abstract

Diversity of possible anatomical variations and configurations of dens invaginatus limits the possibility to conduct its complex treatment in a fully standardized protocol manner. Thus, the objective of the present case report was to approbate targeted approach to Type II dens invaginatus treatment, which may also be interpreted as part of a sequential management strategy with the intention to keep the vitality of a pulp within the main root canal in the first place. The present case report also opens a topic for discussion if “partial success” definition may be applicable for assessment of Type II dens invaginatus treatment outcomes. A 16‐year‐old patient referred to the dentist complaining of painful percussion in the area of Tooth 12. Clinical examination methods (visual examination, sensitivity testing, percussion, and probing) were used for the diagnostics of problematic Tooth 12. To confirm the diagnosis of dens invaginatus, Type II CBCT examination was held. The following treatment was provided: root canal treatment of the invaginated canal only in the first place, while root canal treatment of the main canal was planned to be provided on demand if needed. From the perspective of the presented case report, targeted endodontic treatment of invagination has resolved all the symptoms from the very beginning; meanwhile, the recurrence of the symptoms after some time was due to the involvement of the main root canal. Sequential management with primary targeted invagination treatment of Oehlers′ Type II dens invaginatus presented in this case report seems to be a possible approach for a carefully selected clinical situation aimed at minimizing the level of iatrogenic intervention; however, in the described case report, the end tooth required full endodontic treatment. Considering the risk of potential endodontic complications, development periodical screening of patients, who have undergone primary targeted invagination treatment, seems to be beneficial and reasonable to provide.

## 1. Introduction

Dens invaginatus (DI) represents a developmental anomaly resulting from enamel organ infolding within the dental papilla, which forms intricate internal morphology manifested by invagination present within hard dental tissues [1–6]. This condition is characterized with considerable anatomical variability and may predispose affected teeth to early pulpal involvement and complex endodontic challenges [2, 4, 5]. The most commonly used classification is that proposed by Oehlers and includes three types of this developmental anomaly. Type I is characterized by an enamel‐lined invagination confined to the crown and not extending beyond the cemento‐enamel junction. Type II extends beyond the cemento‐enamel junction into the root as a blind sac and may communicate with the pulp. Type III represents the most severe form, in which the invagination penetrates through the root and exits laterally (Type IIIA) or through the apical foramen (Type IIIB), usually without direct communication with the main pulp chamber [2, 4, 5]. Accurate classification of DI is essential for appropriate diagnosis, treatment planning, and prognosis assessment [1, 2, 5].

Several attempts were made to develop strict treatment guidelines as well as protocoled management strategies for clinical cases of DI considering the clinical and radiological specifics of the pathology [1–5]. Based on the available clinical considerations and formulated treatment recommendations, case selection criteria were proposed which might be implemented into routine clinical practice and could be used for treatment planning to optimize further management process and prognosis of the outcomes [1–6]. Nevertheless, the diversity of possible anatomical variations and configurations of DI limits the possibility of fulfilling their complex treatment in a fully standardized protocol manner, especially in those cases where different treatment approaches may be applicable for the main root canal and invaginated part [7–11]. Taking into account the data from available clinical case reports and case series, one may presume that, even though the relevant clinical guidelines and treatment selection criteria are of great practical significance, DI treatment is still case‐sensitive and should be patient‐oriented [7, 8, 10, 11].

If considering only the invaginated canal treatment option is applicable, then DI treatment success is the aspect, which is not fully resolved from the present evidence‐based perspective [2,7,9,12,13]. There is still no argumentative data available for the evaluation of endodontic treatment success in cases of targeted root canal therapy of the invagination with no intentional interventions within the main root canal [2, 7, 9, 13]. In the vast amount of clinical situations, such an endodontic approach, which may be provided in a preventive manner or due to the causative clinical symptoms, later could be associated with pathological affection of the main root canal that was not curated during the primary treatment session [9–13]. The previous retrospective study reported 11.3% failure rate of the targeted invagination treatment in cases of DI, but what is more important, no grading was proposed for interpretation of timing criteria (the period between finished targeted treatment of invagination and the occurrence of complications within the main root canal), which may be used for classification of primary targeted interventions as either successful or unsuccessful [9].

The present case report demonstrates the outcome of the targeted Oehlers′ Type II DI treatment, highlights the importance of further periodical monitoring of such cases due to the high risk of subsequent endodontic complication development, and opens the topic for discussion if “partial success” definition may be applicable for such clinical cases.

The prevalence of Type II DI tends to vary in the range of 6.25%–26.6% among all identified DI cases, and it depends on such factors as studied population, type of tooth, age, and method of investigation [10–13].

DI Type II cases may remain nonsymptomatic until pulp involvement, while clinically such anomaly might be presented by a palatal groove or pit and some abnormalities in crown morphology (cone‐shaped, dilatation, and talon cusp) or changes of its size (a microdontic teeth) [1]. Treatment options for Type II DI can be either preventive or interventional, while the latter usually takes place after pulp infection and always includes root canal treatment (RCT) [1–4, 9, 14]. The preventive approach involves preventive filling of the grooves and fissures with different materials, including MTA, while RCT may be conducted individually for the invaginated canal, or for both the invaginated and main canal with or without potential removal of invagination [1–4, 9, 14].

## 2. Case Report

A 16‐year‐old patient was referred to the dentist complaining of painful percussion in the area of Tooth 12. The patient reported that previously he had visited two different dental specialists with the abovementioned pain problem, but none of them could help him, while one had referred him to the author of the present case report. In the process of the dental anamnesis data recording, the patient mentioned the history of gingival swelling and the presence of a fistula in the projection of Tooth 12 2 days before referral. In the last 2 days, the fistula has been resolved spontaneously.

Clinical examination provided on 12th of September 2024 revealed that the percussion of Tooth 12 was mildly painful. The cold test revealed a positive reaction, which was slightly reduced when compared with reactions registered at the area of adjacent teeth. Visually, the author noted a small invagination on the palatal side of the tooth crown (Figure 1).

**Figure 1:**
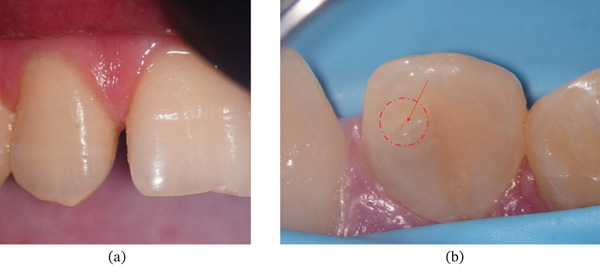
Invagination presented on the palatal side of Tooth 12: (a) view of Tooth 12 from the vestibular side; (b) view of Tooth 12 from the palatal side (red circle and arrow show projection of invagination on the palatal side of crown).

The probing of the tooth surface was normal, even though the author probed the invagination zone very closely. Since the invagination probing was within the normal range, the tooth was examined for the presence of a possible entrance into it, which was subsequently found mesially from the invagination. CBCT examination has been done to gain comprehensive data for differential diagnostics. CBCT data (Planmeca ProMax 3D Classic, Planmeca OY, Helsinki, Finland; technical specifications: anode voltage 60–90 kV, anode current 1–14 mA, focal spot 0.5 mm, fixed anode, image detector—flat panel, maximum volume with a single scan Ø8 × 8 cm, and voxel size 200 *μ*m) confirmed DI presence, which, according to the x‐ray criteria (a blind sack within the root boundaries without communication with the main pulp chamber), could be categorized as Type II according to the Oehlers′ classification (Figure 2).

**Figure 2:**
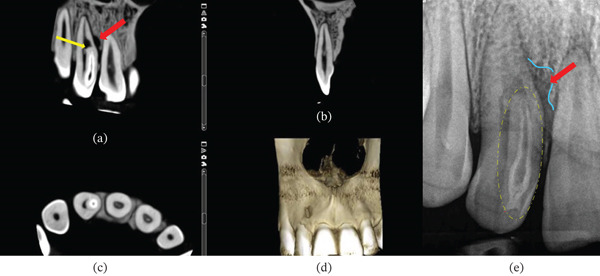
CBCT and periapical x‐ray images demonstrating the presence of Oehlers′ Type II dens invaginatus: (a) coronal CBCT cross‐section (red arrow shows projection of bone rarefication adjacent to projection of invagination; yellow arrow shows the deepest portion of invagination with no visible connection with main root canal at least based on present CBCT resolution); (b) sagittal CBCT cross‐section; (c) axial CBCT cross‐section; (d) 3D rendering of jaw fragment; (e) periapical x‐ray (yellow contour highlights the projection of the invagination, while the red arrow and blue line indicate the area of periapical bone rarefaction adjacent to the invagination).

Considering the history of a previously resolved sinus tract, tenderness to percussion, positive but diminished response to cold sensibility testing, and radiographic evidence of bone rarefaction observed on both periapical radiography and CBCT, the diagnostic findings were considered equivocal. Positive response to cold testing suggested the presence of residual vital pulp tissue, whereas the apical findings were indicative of apical inflammation. In accordance with the American Association of Endodontists terminology, the apical diagnosis was established as symptomatic apical periodontitis. However, due to the complex anatomy associated with Type II DI and the possibility of differential involvement of the invaginated canal and the main canal pulp space, the pulpal status could not be determined with complete certainty based on conventional diagnostic testing alone.

According to established clinical guidelines and the available diagnostic findings, conventional RCT of both the main root canal and the invaginated canal represented the most predictable therapeutic option. However, CBCT examination demonstrated a Type II DI with a distinct invaginated canal, while pulp sensibility testing yielded equivocal rather than definitively negative results. Furthermore, the entrance to the invagination was clinically identifiable, raising the possibility that the pathological process could be confined primarily to the invaginated structure without complete involvement of the main pulp space. Based on these findings, an additional alternative treatment option was proposed, which was focused on the hypothesis of only invaginated canal being pathologically involved. Considering this, the following treatment options were proposed to the patient: (1) RCT of both the main root canal and the invaginated one and (2) RCT of the invaginated canal only (with the possibility to conduct RCT of the main canal later if symptoms persist). The second option has been called a targeted approach or conservative treatment in terms of trying to keep the vitality of the pulp within the main root canal. Nevertheless, the patient was informed that this strategy was associated with a higher probability of requiring future treatment of the main canal compared with immediate treatment of both canal systems. After discussion of the expected benefits, risks, and uncertainties associated with each option, informed consent was obtained, and treatment of the invaginated canal alone was performed. The patient′s main argument to choose the second option was personal intention to go with a biologically conservative approach aimed at potentially preserving pulp vitality within the main root canal whenever possible.

Endodontic treatment of the invaginated canal was provided on the same date as clinical examination (on 12th of September 2024). Ubistesin solution 4% with epinephrine 1:100000 (3M, Deutschland GmbH, Germany) was used for the infiltration anesthesia. The tooth was isolated using a rubber dam prior to initiation of endodontic treatment. Access to the invaginated canal was obtained under magnification using a diamond bur, while conventional access refinement was performed using Endo‐Z burs (MANI, Inc., Tochigi, Japan). Canal patency was assessed with a size SANI K‐file of Size 10/0.02 (Chengdu Sani Medical Equipment Co., Chengdu, China). Working length was determined using an electronic apex locator and verified radiographically. The mechanical treatment of the invaginated root canal has been done with a manual SANI K‐file of Size 10/0.02 (Chengdu Sani Medical Equipment Co., Chengdu, China) (Figure 3).

**Figure 3:**
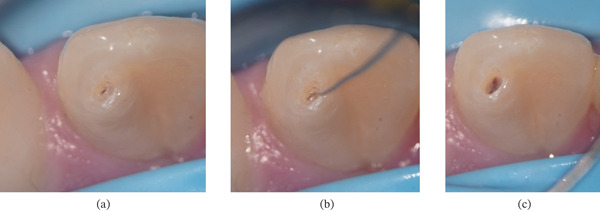
The access point and the mechanical treatment of dens invaginatus: (a) the access point; (b) initiation of the mechanical treatment process; (c) view after mechanical treatment.

The periapical x‐ray image confirmed the negotiation of the invaginated root until the end, while an apex locator had not registered the presence of any apical foramen in the invaginated root. Further instrumentation of the root canal was provided with SANI Blue Storm files up to the size of 30/0.04 (Chengdu Sani Medical Equipment Co., Chengdu, China). Irrigation was performed with 5.25% sodium hypochlorite solution (Chloraxid 5.25%, CERKAMED, Stalowa Wola, Poland) delivered with a side‐vented irrigation needle positioned short of the working length. Ultrasonic activation was performed using an ultrasonic activator (COXO ULTRA SMART Endo, Foshan COXO Medical Instrument Co., Ltd., Foshan, China) for three activation cycles of 20–30 s each. Following completion of sodium hypochlorite irrigation, the canal was rinsed with 17% EDTA (ENDO‐SOLUTION, CERKAMED, Stalowa Wola, Poland) for 1 min to facilitate smear layer removal.

Sodium hypochlorite and EDTA were not used simultaneously. After completion of sodium hypochlorite irrigation, the canal was flushed with sterile saline before application of 17% EDTA. Following EDTA irrigation, a final rinse with sodium hypochlorite was performed prior to canal drying and obturation.

After completion of irrigation, the canal was dried using sterile paper points until complete dryness was achieved. RCT of the invaginated canal was completed in a single clinical session, and the canal was subsequently obturated. Obturation was performed with gutta‐percha and AH Plus sealer (DENTSPLY Maillefer, Tulsa, United States) using a continuous wave of condensation as an obturation technique utilizing C‐Fill COXO equipment (Foshan COXO Medical Instrument Co., Ltd., Foshan, China) (Figures 4 and 5).

**Figure 4:**
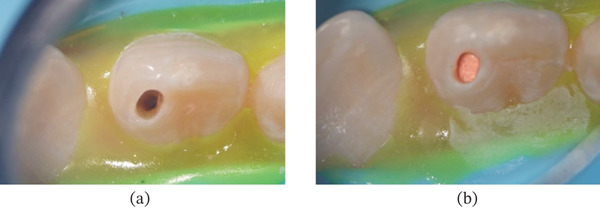
Dens invaginatus endodontic treatment: (a) view after finished root canal instrumentation and irrigation; (b) view after obturation of invaginated canal.

**Figure 5:**
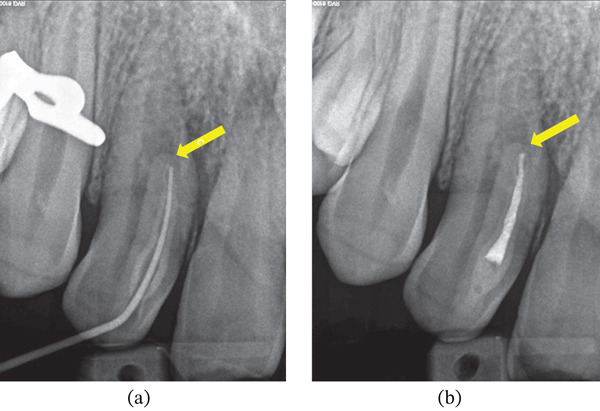
X‐ray control of dens invaginatus endodontic treatment: (a) x‐ray control of gutta‐percha point fit; (b) x‐ray control of obturation quality (yellow arrows demonstrate the deepest portion of invagination).

The entire treatment procedure described above was performed in a single appointment. Access cavity preparation, canal negotiation, working length determination, chemomechanical preparation, irrigation, drying, and obturation of the invaginated canal were completed during the same clinical session.

As soon as the invaginated root canal had been obturated, the access point was immediately restored with light‐cured composite material Estelite Tokuyama (Tokuyama Dental Corporation, Tokyo, Japan) with implementation of total‐etch adhesive protocol with Optibond FL (KERR, Kloten, Switzerland). The patient was informed that the main root canal might be devitalized, and a second intervention would be required if specific symptoms occurred. One week after the treatment on the 19th of September 2024, the patient came for a follow‐up visit and reported that all painful sensations had been resolved, and the tooth did not disturb him anymore.

However, 3 months later, on the 22nd of December 2024, the patient was referred to our clinic again with the complaint of a spontaneous aching pain in Tooth 12, which has been aggravated at night or while eating a hot meal. Clinical re‐examination revealed that the composite restoration in the area of previously presented invagination has not been compromised, and no fistula signs were presented in the projection of the gingiva. The percussion was negative as well as the probing was within the normal range. The cold test revealed a delayed, weakened reaction. The diagnosis of irreversible pulpitis of Tooth 12 has been made, and the option of endodontic treatment was offered.

Treatment of the main root canal was started on the 22nd of December 2024. Ubistesin solution 4% with epinephrine 1:100000 (3M, Deutschland GmbH, Germany) was used for the infiltration anesthesia. The pulp chamber was exposed with dental burs, and a small volume of purulent masses has been removed. Due to the use of an operating microscope at all stages of the treatment, the vital condition of the pulp was noted starting from the middle third of the root. Abundant irrigation of the root canal was performed with 5.25% sodium hypochlorite solution (Chloraxid 5.25%, CERKAMED, Stalowa Wola, Poland) along with supportive ultrasonic activation. The working length of 26 mm was determined with the help of a manual K‐file of Size 15/0.02 (Chengdu Sani Medical Equipment Co., Chengdu, China) and apex locator. The instrumentation was done with Ni‐Ti rotary SOCO SC files (Chengdu Sani Medical Equipment Co., Chengdu, China) till the final size of 45/0.04. The smear layer was removed with 17% EDTA solution (ENDO‐SOLUTION, CERKAMED, Stalowa Wola, Poland). Due to the anatomical complexity, it was decided to manage the tooth using calcium hydroxide as an intracanal dressing for a period of 2 weeks to improve the treatment outcome. Usage of calcium hydroxide as an intracanal dressing was considered in the process of the main canal treatment since the access to the canal zone right under the invagination (anatomical undercut) was complicated, as well as due to the diagnosed pulp necrosis, the evidence of which was the debrided purulent content. Calcium hydroxide intracanal dressing has not been used during the treatment of an invaginated canal because of a simple and straight anatomy of the latter as well as because of no signs of pulp necrosis during the initial patient referral. Two weeks later, the patient reported a complete resolution of the symptoms.

During the next visit held on 5th of January 2025, an analogue of GentleFile GF‐brush (MedicNRG, Kibbutz Afikim, Israel) was applied to extract calcium hydroxide from the endodontic space following manufacturer′s recommendations. However, an x‐ray control image performed after canal instrumentation revealed a significant amount of calcium hydroxide residuals in the area of dilatation under the invagination. A subsequent passive ultrasonic irrigation (PUI) was carried out with the use of sodium hypochlorite and EDTA solutions to clean out all the calcium hydroxide residuals. A control radiograph taken to evaluate the fitting of the gutta‐percha point confirmed a complete absence of calcium hydroxide residuals. Obturation was performed with a gutta‐percha point and AH Plus sealer (DENTSPLY Maillefer, Tulsa, United States) using a continuous wave of condensation as an obturation technique utilizing C‐Fill COXO equipment (Foshan COXO Medical Instrument Co., Ltd., Foshan, China) (Figures 6 and 7).

**Figure 6:**
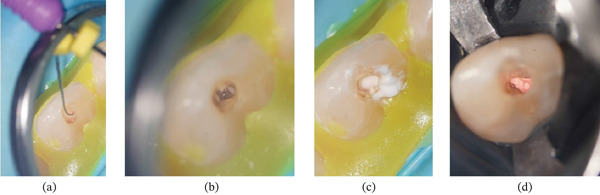
The clinical stages of the main root canal endodontic treatment: (a) negotiation; (b) view after root canal instrumentation; (c) view after applying Ca(OH)_2_‐based agent as intracanal dressing; (d) view after obturation.

**Figure 7:**
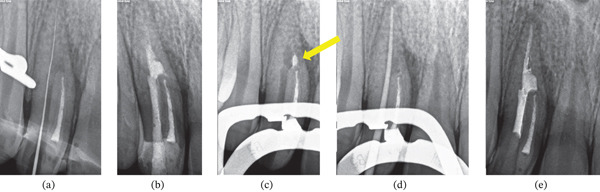
Periapical x‐ray control image of the main root canal: (a) checking the working length; (b) after applying Ca(OH)_2_‐based agent as an intracanal dressing; (c) view after applying GentleFile GF‐brush to extract calcium hydroxide from the endodontic space (there are residuals of Ca(OH)_2_‐based agent in the root canal highlighted with yellow arrow); (d) checking the fitting of gutta‐percha point; (e) view after obturation.

All the endodontic treatment was provided with the use of an operating microscope, considering such approach as a standard of care within endodontic practice, especially in complex cases like DI [15, 16]. Operative microscope C‐CLEAR 2 Pro (COXO Medical Instrument Co., Ltd, Foshan, China) with parameters of apochromatic optical system, continuous vario‐zoom of 0.4×–2.5×, total magnification range of 1.6×–19×, and field of view in the range 11.5–138.4 mm was used at the following stages of procedure: while forming access to the invaginated canal and later while forming access to the main canal, during instrumentation stage to assure correct trajectory of endodontic instrument passage, while also to visually assess cleanliness of the root canal at the end of instrumentation stage, and also at the stage of checking completeness of removing calcium hydroxide from root canal during the second stage of main RCT. Low magnification levels (2×–5×) were used for access cavity preparation and orientation within the pulp chamber, while a high magnification level (19×) was used to assess the apical region of the invaginated canal regarding its potential communication with the main root canal.

Smear layer at the crown part of the tooth was removed by the air abrasion method with aluminum oxide particles. The tooth was restored by Estelite Tokuyama light‐curing material (Tokuyama Dental Corporation, Tokyo, Japan) along with utilizing the total‐etch adhesive protocol with Optibond FL (KERR, Kloten, Switzerland).

The patient came for a follow‐up examination 11 months after the provided treatment (17th of November 2025). No complaints or pathological clinical signs were presented, while CBCT examination also revealed a healthy condition of periapical tissues (Figure 8).

**Figure 8:**
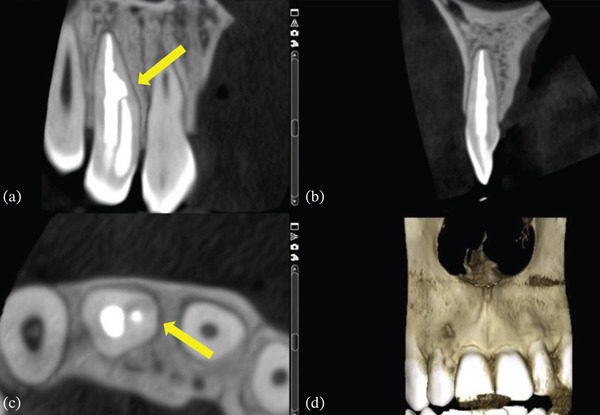
CBCT image 11 months after the provided treatment with no signs of pathological periapical changes present: (a) coronal CBCT cross‐section (no bone rarefication noted in the projection of invagination marked with yellow arrow); (b) sagittal CBCT cross‐section; (c) axial CBCT cross‐section (no bone rarefication noted in the projection of invagination marked with yellow arrow); (d) 3D rendering of jaw fragment.

A timeline of clinical events provided during the management of the abovementioned case report is presented within Figure 9.

**Figure 9:**
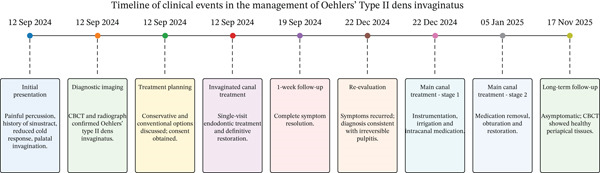
Timeline of clinical events provided during the management of Oehlers′ Type II dens invaginatus with primary targeted invagination treatment.

Written informed consent was obtained from the patient for treatment, clinical documentation, and publication of anonymized clinical data and radiographic images included in this report.

## 3. Discussion

The presented clinical case demonstrated sequential management with primary targeted invagination treatment of Type II DI treatment, which may also be interpreted as conservative one considering the objective to keep the vitality of the pulp within the main root canal in the first place.

Oehlers′ Type II DI is reported to be the second (or, by some literature data, even first) most prevalent type of dens in dente [17–20]. Even though in previous research, the general prevalence of DI was estimated at the level of 0.04%–10% [1, 2]; in a recent retrospective study, the prevalence rate reached 26% among studied tooth samples and 37% among involved patients′ samples [17]. Usage of cone‐beam computed tomography supports higher identification frequency of DI compared to periapical x‐ray images and conventional or rendered panoramic images [21]. Moreover, a few cone‐beam computed tomography studies reported a higher prevalence rate of Oehlers′ Type II DI compared to Type I and Type III [19, 20].

In the presented case report, the usage of CBCT for examination purposes of the problematic tooth helped to identify DI Type II as per Oehlers′ diagnostic criteria [7, 22]. The following includes the presence of a blind sac lined with enamel within the root boundaries, which may or may not have communication with the main pulp chamber [22]. In the recent case series, modifications to the Oehlers′ classification were proposed associated with distinguishing subtypes of Type II depending on the extension of invagination into the root (IIA, to the coronal third of the root; IIB, to the middle third of the root canal; and IIIC, to the apical third of the root canal) [8]. The authors elaborated such differentiation of Type II DI based on the possibility to implement different successful treatment modalities considering the extension of the invagination. Considering proposed modifications to the Oehlers′ classification, DI described in this case report may be classified as Type II (due to the Oehlers′ classification [7, 22]) and Subtype IIB (due to the modified classification proposed by Kritika et al. [8]).

Regardless of the type of DI, the usage of CBCT is essential for the objective evaluation of anatomical configuration and further planning of the treatment algorithm [23]. Recent updates represented in the AAE and AAOMR Joint Position Statement regarding the use of cone‐beam computed tomography in endodontics highlight the significance of 3D root canal morphology assessment while using limited FOV CBCT as preferred imaging modality [24]. Considering the present progress in machine learning technologies implementation into the clinical dental practice, further usage of artificial intelligence‐based tools may be helpful in establishing not only anatomical features of root morphology but also at supporting clinical decision‐making for complex cases, like DI, using obtained CBCT results [25–27].

Differentiation of DI type according to the proposed Oehlers′ classification and its modifications can be used as guidance during future therapeutic interventions, and it might help to implement the most sufficient treatment protocol. In the present case report, CBCT helped to verify the absence of communication between the main canal and the invaginated one, which also was used as an argument to carry out endodontic treatment only within the invagination.

State‐of‐the‐art literature reviews summarized the following treatment options for Type II DI: preventive filling, filling of invagination with MTA, endodontic treatment of only the main canal, endodontic treatment of only the invaginated canal, invagination removal with further endodontic treatment, apexification of the main canal with Ca(OH)_2_‐based agent, apexification of the main canal with MTA, endodontic treatment supported by periapical surgical intervention, and pulp revascularization of the main canal [1, 2]. The preference for any of the abovementioned treatment techniques depends on the established condition of pulp vitality, caries status, and formation level of the apical foramen [1, 2, 4–6]. Available approaches to conservative treatment of only invaginated canal, which are presented in the literature, differ by their procedural aspects and level of intervention [9, 14, 21, 28, 29]. The provision of conservative treatment for DI Type II is based on the possibility to retain pulp vitality within the main canal in case the pulp infection occurs within the invaginated canal only. Except for the targeted treatment approach described in this case, the specific decision‐making process for Type II DI treatment is already well described in a number of publications with consideration of the differential impact of various influential factors [1–4].

So far, only one study presented the retrospective success rate of preventive treatment for DI Type II cases with a 13.4% tooth‐based failure rate (specifically for Type II) and 15.8% patient‐based failure rate [9]. Among all nine problematic teeth noted in the abovementioned study, five of them presented complications within the first year of monitoring after conservative invagination treatment, one during the second year of monitoring, two during the third year of monitoring, and one during the sixth year of monitoring [9]. Even though authors have classified all these cases as treatment failures, it is worth mentioning that in the majority of cases, endodontic treatment of the main canal helped to resolve pathological symptoms [9].

In the present case, symptom recurrence occurred 3 months after treatment limited to the invaginated canal. From a conventional endodontic perspective, such an outcome cannot be interpreted as unequivocal treatment success. The precise mechanism responsible for recurrence remains unknown. One possible explanation is the presence of anatomical communication between the invaginated canal and the main root canal system that was not identifiable on the available CBCT images. Previous studies have reported that complex internal anatomy associated with DI may include accessory communications that are difficult to detect radiographically [3]. If such communication existed in the present case, it could theoretically have facilitated microbial exchange between the two compartments. However, this hypothesis cannot be verified based on the available clinical and radiographic data and should therefore be interpreted with caution. Alternative explanations, including undetected pre‐existing involvement of the main pulp space or progression of pathology independent of direct canal communication, cannot be excluded.

The present case illustrates a potential challenge in interpreting treatment outcomes following targeted management of Type II DI. From a theoretical and philosophical perspective, the present case highlights the possibility that treatment outcomes in sequentially managed Type II DI cases may not always fit neatly into conventional binary classifications of success and failure. Specifically, targeted treatment of the invaginated canal may initially achieve symptom resolution and preservation of vitality within the main root canal system, while subsequent pathology may necessitate additional intervention. Such a clinical course raises broader questions regarding whether outcome assessment should focus exclusively on the initial procedure, the entire treatment pathway, or the final long‐term condition of the tooth. The present report does not attempt to answer these questions or propose alternative outcome classifications. Rather, it is intended to stimulate future discussion and research regarding the interpretation of staged treatment outcomes in anatomically complex cases of DI.

Although one could argue that the initial intervention achieved a degree of short‐term therapeutic benefit, the authors acknowledge that current endodontic outcome classifications do not include a category equivalent to “partial success.” Such a category of “partial success” should be regarded only as a hypothesis‐generating clinical consideration rather than evidence supporting modification of existing success criteria. Further prospective studies involving larger cohorts would be necessary to determine whether such cases represent a distinct clinical outcome category or remain adequately addressed within current endodontic outcome frameworks. But in the manner of hypothesis proposal and philosophical discussion, outcomes obtained in cases of sequential management with primary targeted invagination treatment of teeth with Type II DI might be interpreted in three possible ways from the endodontic success perspective:

1. As a failure: if conservative treatment of invagination considered as a treatment endpoint.
2. As a partial success: if conservative treatment of invagination considered as an intermediate point of the treatment in a short‐term perspective, when recurrent symptoms might be managed by means of the endodontic treatment of the main canal.
3. As a success: if conservative treatment of invagination considered as an intermediate point of treatment in a long‐term perspective, when recurrent symptoms might be managed by means of the endodontic treatment of the main canal.

Even though the definition of “partial success” is lacking ground scientific evidence in present time and it could not be classified at either of well‐established categories of “success” (healed outcome), “failure” (not healed outcome), or “healing” (in progress), present case report represents methodological attempt to argument relevance of partial success definition at least for cases of DI Type II treatment in condition of only invaginated canal being primarily affected.

The important question regarding the differentiation of partial success and success, which cannot be resolved due to the deficiency of evidence‐based data, is what time interval can be interpreted as short‐ and long‐term perspective during the assessment of outcomes obtained after conservative Oehlers′ Type II DI treatment. Considering data from Ridell et al.′s retrospective study [9], which demonstrated that the most complications after preventive invagination treatment occurred during the first year of monitoring, a period of 1 year may be proposed to be used as a time criterion to distinguish partial success and success during conservative treatment of Oehlers′ Type II DI. It should be highlighted that the proposed threshold of 1 year to distinguish cases with partial success and success is hypothesis‐generated, derived primarily from retrospective evidence obtained in a previous study [9] and such could not be established as an evidence‐based benchmark. However, in the future, such a threshold may be considered to accumulate and categorize a greater amount of data regarding outcomes of treatment provided within Type II DI cases.

The authors fully acknowledge that abovementioned considerations are entirely speculative and do not represent a validated proposal for modification of existing endodontic outcome classifications. Current success criteria remain based on well‐established clinical and radiographic parameters supported by considerably stronger evidence than can be derived from a single‐case report. Nevertheless, the present case highlights the complexity of outcome assessment in anatomically challenging conditions such as Type II DI and may serve as a basis for future discussion regarding the interpretation of staged treatment outcomes in such cases.

Conservative, or so‐called targeted treatment of Oehlers′ Type II DI presented in this case report, seems to be a perspective approach aimed at minimizing the level of iatrogenic intervention; nevertheless, further in‐detail studies are needed to evaluate its objective level of success and risk of complications before recommending it for all cases of Type II DI in routine clinical practice. An important consideration when interpreting the present case is the comparison between the selected treatment strategy and conventional management involving initial treatment of both the invaginated canal and the main root canal system. From a predictability perspective, treatment of both canal systems would likely be regarded as the more conventional approach, as it reduces the possibility of future pathology arising from undetected involvement of the main pulp space and may decrease the likelihood of requiring subsequent intervention. The recurrence of symptoms observed in the present case after 3 months may be interpreted as a limitation of the vitality‐preserving strategy.

Conversely, the primary potential advantage of targeted treatment of the invaginated canal is the possibility of preserving vitality within the main root canal system when definitive evidence of its pathological involvement is lacking. Such an approach may be viewed as biologically conservative; however, the potential benefit of vitality preservation must be balanced against the increased uncertainty regarding long‐term outcomes. The present case should therefore not be interpreted as supporting superiority of one strategy over another but rather as illustrating the clinical trade‐offs associated with different treatment philosophies in anatomically complex cases of Type II DI.

There is a lack of structured statistical data regarding the success of different approaches already provided for Type II DI treatment which could be used for the comparison, but available publications demonstrate a general trend of endodontic treatment provided for both invaginated and main canals with or without removal of invagination being more successful compared to other treatment options. However, such an approach is a more extensive intervention compared to proposed conservative management, while the latter is aimed at retaining tooth vitality for the possible longest time.

Interpretation of the available literature regarding DI should be undertaken with caution. Most published evidence consists of individual case reports and small case series, which provide valuable clinical insights but represent a relatively low level of evidence [9, 14, 15, 21, 28, 29]. In addition, publication bias may influence the available literature, as unusual presentations and favorable treatment outcomes are more likely to be reported than unsuccessful cases or routine clinical experiences. Consequently, the true predictability of different treatment approaches may be difficult to estimate from the existing evidence.

Another important limitation is the lack of comparative clinical studies directly evaluating alternative management strategies for Type II DI. As a result, uncertainty remains regarding the optimal balance between conservative vitality‐preserving approaches and more comprehensive endodontic treatment strategies. Therefore, treatment decisions continue to rely heavily on individual anatomical characteristics, clinical judgment, and patient‐specific considerations. The present case should be interpreted within this context and viewed as a clinical observation rather than evidence supporting a particular treatment philosophy or standard of care.

The presented case report has several limitations, one of which is related to single‐case observation, so the findings cannot be generalized to all DI presentations. Although CBCT imaging and magnification were used, microanatomical communications cannot be completely excluded in the present case, which may impact the initial decision for treatment strategy. Furthermore, the follow‐up period of 11 months represents midterm observation, and longer term monitoring is required to confirm stability of periapical healing. Future prospective studies with larger cohorts are necessary to validate the proposed treatment strategy, but it should be highlighted that the targeted invagination‐only approach may be interpreted as an individualized clinical decision rather than a universally recommended protocol. It is important to acknowledge that the implemented treatment strategy of targeted intervention diverged from established endodontic guidelines; however, this approach was implemented with a patient‐centered rationale, taking into account the anatomical complexity of the case, the variability of diagnostic findings, and the intention to preserve pulp vitality whenever possible. Nevertheless, it also may be highlighted that analogical approach was already described within the literature, representing different levels of success obtained [9, 14, 21, 28, 29].

Although the patient actively participated in the treatment decision‐making process and provided informed consent after discussion of alternative treatment options, the potential patient‐centered advantages or disadvantages of the selected treatment strategy cannot be objectively assessed based on the available data. Future studies incorporating patient‐reported outcomes and preference‐based measures would be valuable for evaluating the broader clinical relevance of vitality‐preserving treatment approaches in DI cases.

Ongoing monitoring of patients who have undergone Type II DI staged treatment seems to be a suitable postoperative strategy in terms of screening for changes related to the development of potential periradicular complications and ensuring that patients at high risk are provided with dental treatment promptly.

The present case also highlights several clinically relevant key learning points of the diagnosis and management of Type II DI. First, pathological involvement of the invaginated canal and the main root canal system may not occur simultaneously, creating considerable diagnostic complexity and potentially influencing treatment planning. In this regard, CBCT imaging represents a valuable adjunctive diagnostic tool for assessment of the internal anatomy and extent of the anomaly, although subtle anatomical communications between canal systems may remain beyond the limits of radiographic detection.

The clinical course observed in this case suggests that, in carefully selected situations, targeted treatment of the invaginated canal may initially result in symptom resolution while preserving vitality within the main root canal system. Nevertheless, the subsequent recurrence of symptoms emphasizes that preservation of vitality does not necessarily eliminate the possibility of future pathological changes. Importantly, such recurrence should not automatically be interpreted as technical failure of the initial procedure, but rather as a reflection of the biological and anatomical complexity associated with DI.

Given these considerations, clinical and radiographic follow‐up may be beneficial after conservative treatment approaches, particularly when preservation of vitality within the main canal is among the treatment objectives. At present, however, the available evidence regarding the optimal management of Type II DI remains limited and is largely derived from case reports and small case series. Consequently, treatment decisions should continue to be individualized according to the specific anatomical characteristics, clinical findings, and patient preferences encountered in each case.

## 4. Conclusion

The proposed approach of sequential management with primary targeted invagination treatment of teeth with Type II DI, which by its essence is aimed at the intention to keep the pulp vitality within the main root canal, may be suitable for carefully selected clinical situations, but so far, it could not be interpreted as superior or equivalent to conventional endodontic treatment of a problematic tooth. Considering the risk of potential endodontic complications, development periodical screening of patients, who have undergone primary targeted invagination treatment, seems to be beneficial and reasonable to provide. Following validation through large‐scale clinical investigations, such treatment modality may provide a rationale for revisiting the conventional success criteria applied to the endodontic management of teeth affected by DI. However, the current evidence base remains limited, and further well‐designed studies are required before any modifications to existing success criteria can be justified.

## Author Contributions

**Igor Noenko:** investigation, conceptualization, research, validation, methodology, visualization, writing—original draft. **Myroslav Goncharuk-Khomyn:** investigation, conceptualization, writing—original draft, writing—review and editing, validation, data curation, formal analysis, visualization, and supervision. **Mariya Kubatska:** conceptualization, writing—review and editing, formal analysis, and validation.

## Funding

No funding was received for this manuscript.

## Disclosure

All authors have read and approved the final version of the manuscript. Myroslav Goncharuk‐Khomyn, as the corresponding author and manuscript guarantor, had full access to all of the data in this study and takes complete responsibility for the integrity of the data and the accuracy of the data analysis. All authors contributed to this paper have been mentioned in the manuscript, and mentioned authors have not asked for any help from any other persons or services during present research or preparation of present manuscript. Mentioned authors have covered all the authorship responsibilities, requirements, and criteria just by themselves, and there is nobody to additionally mention in the Acknowledgments section.

## Ethics Statement

The study was conducted in accordance with the Declaration of Helsinki and approved by the Institutional Review Board of Faculty of Dentistry at Uzhhorod National University (Protocol Code 10/2025 dated by 01/03/2025). This case report was prepared and reported in accordance with the CARE (CAse REport) reporting guidelines. As this report describes a single anonymized clinical case managed according to routine standards of care, formal ethical committee approval was not required.

## Consent

Written informed consent for treatment, clinical documentation, and publication was obtained from the patient.

## Conflicts of Interest

The authors declare no conflicts of interest.

## Data Availability

Data supporting this case report is available from the corresponding author on reasonable request.
